# Assessing cardiorespiratory interactions via lagged joint symbolic dynamics during spontaneous and controlled breathing

**DOI:** 10.3389/fnetp.2023.1211848

**Published:** 2023-08-03

**Authors:** Beatrice Cairo, Vlasta Bari, Francesca Gelpi, Beatrice De Maria, Alberto Porta

**Affiliations:** ^1^ Department of Biomedical Sciences for Health, University of Milan, Milan, Italy; ^2^ Department of Cardiothoracic, Vascular Anesthesia and Intensive Care, IRCCS Policlinico San Donato Milanese, Milan, Italy; ^3^ IRCCS Istituti Clinici Scientifici Maugeri, Milan, Italy

**Keywords:** coupling strength, phase relationship, nonlinear interactions, heart rate variability, respiratory sinus arrhythmia, cardiac control, autonomic nervous system

## Abstract

**Introduction:** Joint symbolic analysis (JSA) can be utilized to describe interactions between time series while accounting for time scales and nonlinear features. JSA is based on the computation of the rate of occurrence of joint patterns built after symbolization. Lagged JSA (LJSA) is obtained from the more classical JSA by introducing a delay/lead between patterns built over the two series and combined to form the joint scheme, thus monitoring coordinated patterns at different lags.

**Methods:** In the present study, we applied LJSA for the assessment of cardiorespiratory coupling (CRC) from heart period (HP) variability and respiratory activity (R) in 19 healthy subjects (age: 27–35 years; 8 males, 11 females) during spontaneous breathing (SB) and controlled breathing (CB). The R rate of CB was selected to be indistinguishable from that of SB, namely, 15 breaths·minute^−1^ (CB15), or slower than SB, namely, 10 breaths·minute^−1^ (CB10), but in both cases, very rapid interactions between heart rate and R were known to be present. The ability of the LJSA approach to follow variations of the coupling strength was tested over a unidirectionally or bidirectionally coupled stochastic process and using surrogate data to test the null hypothesis of uncoupling.

**Results:** We found that: i) the analysis of surrogate data proved that HP and R were significantly coupled in any experimental condition, and coupling was not more likely to occur at a specific time lag; ii) CB10 reduced CRC strength at the fastest time scales while increasing that at intermediate time scales, thus leaving the overall CRC strength unvaried; iii) despite exhibiting similar R rates and respiratory sinus arrhythmia, SB and CB15 induced different cardiorespiratory interactions; iv) no dominant temporal scheme was observed with relevant contributions of HP patterns either leading or lagging R.

**Discussion:** LJSA is a useful methodology to explore HP–R dynamic interactions while accounting for time shifts and scales.

## Introduction

Cardiorespiratory coupling (CRC) is defined as the interaction between cardiac and respiratory rhythms. CRC covers a variety of phenomena independent of each other (Penzel et al., 2016; Elstad et al., 2018). Respiratory sinus arrhythmia (RSA) is the most studied effect of CRC (Hirsch and Bishop, 1981; Eckberg, 1983; Hayano et al., 1996). RSA accounts for the periodical modification of heart period (HP) with respiratory activity (R) resulting from HP shortening during inspiration and HP lengthening during expiration under physiological conditions and breathing rates. RSA is a frequency-dependent phenomenon (Angelone and Coulter, 1964; Hirsch and Bishop, 1981; Eckberg, 1983; Brown et al., 1993), but it is not affected by voluntary control of breathing (Patwardhan et al., 1995; Pinna et al., 2006). Another phenomenon under the umbrella of CRC is cardiorespiratory phase synchronization, which takes the form of short intermittent epochs of stable occurrence of heartbeats at specific phases of the respiratory cycle (Schäfer et al., 1998; Schäfer et al., 1999; Bartsch et al., 2012; Kuhnhold et al., 2017; Mazzucco et al., 2017; Cairo et al., 2021; Ottolina et al., 2023). Cardiorespiratory phase synchronization has been observed in athletes (Schäfer et al., 1998; Cairo et al., 2021), healthy subjects (Schäfer et al., 1999; Bartsch et al., 2012), patients in intensive care units (Mazzucco et al., 2017; Ottolina et al., 2023), and post-infarction patients (Kuhnhold et al., 2017). Cardioventilatory coupling, defined as a stable temporal relationship between the onset of the inspiration and the last heartbeat preceding it (Galletly and Larsen, 1997; Tzeng et al., 2003; Larsen et al., 2010; Friedman et al., 2012), is another form of CRC independent of RSA and cardiorespiratory phase synchronization.

Therefore, it is clear that the cardiac and respiratory systems interact in complex and multifaceted ways, including various nonlinear types of interplay (Elstad et al., 2018; Abreu et al., 2023), and this influence might be mediated by the baroreflex as well (Dick et al., 2005; Abreu et al., 2020). As such, many bivariate methodologies that are capable of accounting for high-order statistical moments have been proposed in the context of CRC assessment (Moser et al., 1995; Galletly and Larsen, 1997; Schäfer et al., 1998; Penzel et al., 2016). Among them, joint symbolic analysis (JSA) was first proposed as an extension of univariate symbolic analysis (Baumert et al., 2002; Wessel et al., 2009; Kabir et al., 2011; Schulz et al., 2013a; Porta et al., 2015a; Porta et al., 2015b) and applied to beat-to-beat HP variability and R for the quantification of CRC in sports, health, and pathology (Kabir et al., 2011; Reulecke et al., 2012; Schulz et al., 2013b; Baumert et al., 2015; Schulz et al., 2015; Reulecke et al., 2018; Schulz et al., 2018; Abreu et al., 2019). The introduction of a time shift between patterns, thus leading to a lagged JSA (LJSA), allows monitoring CRC with different temporal schemes and latencies (Wessel et al., 2009; Suhrbier et al., 2010; Kabir et al., 2011; Baumert et al., 2015). Because some JSA tools can account for the time scales when analyzing interactions by classifying joint symbolic schemes composed of patterns featuring different frequency contents (Porta et al., 2015b; Bari et al., 2016; Porta et al., 2016), LJSA could be made scale-specific.

The temporal scheme in HP–R dynamic interactions remains poorly elucidated. Modeling approaches describe cardiorespiratory interactions because of the exogenous actions of R on HP (Baselli et al., 1994; Triedman et al., 1995; Porta et al., 2000a; Chen and Mukkamala, 2008; Porta et al., 2012a; Porta et al., 2013a). This hypothesis was followed even when applying JSA, given that in Kabir et al. (2011), the delay of HP to R was optimized, while advancements of HP to R were not considered. However, the leading action of the heart to the respiratory system has been identified (Galletly and Larsen, 1997; Galletly and Larsen, 1999) and confirmed in healthy individuals (Tzeng et al., 2003; Larsen et al., 2010; Friedman et al., 2012). This link was supported via cross-spectral analysis, especially at breathing rates below 0.15 Hz, while in the range of respiratory rates between 0.15 to 0.25 Hz, heart rate fluctuations and R volume appear to be in phase (Saul et al., 1989). More sophisticated parametric modeling approaches corroborate the finding that HP might lead R (Yana et al., 1993; Perrott and Cohen, 1996; Porta et al., 2013b). This effect is compatible with the fastness of cardiac neural control compared to the slowness of the thoracic movements (Yana et al., 1993; Triedman et al., 1995; Perrott and Cohen, 1996; Porta et al., 2013b). However, some studies provided a less trivial interpretation involving the action of a latent confounder, such as the baroreflex (Abreu et al., 2020) triggering the onset of inspiration (Galletly and Larsen, 1999; Dick et al., 2005). We tested the hypothesis that the association between heartbeat and R could be higher at specific time shifts and that this result might depend on breathing rate.

The aim of the present study is the evaluation of the HP–R association in healthy subjects during SB and CB using an LJSA approach capable of differentiating interactions at different time scales. The rate of CB is selected to be indistinguishable from that of SB, namely, at 15 breaths·minute^−1^ (CB15), or significantly slower, namely, 10 breaths·minute^−1^ (CB10) but above the limit of 0.15 Hz. The protocol includes CB10 and CB15 because both paced R rates are in the range of R frequencies leading to very fast, in-phase modifications of heart rate and R volume (Saul et al., 1989), even though interactions occur at significantly different time scales that are slower in the case of CB10 than CB15. Accordingly, we tested a physiological range of lags, accurately chosen according to the type of collected signals, to explore variations of the HP–R association with time shift. The link of markers derived from LJSA and coupling strength was tested over simulations, and surrogate data were exploited to check the significance of interactions. Preliminary results were presented at the 12th conference of the European Study Group on Cardiovascular Oscillations (Cairo et al., 2022).

## LJSA

The two series $HP=\left\{{HP}_{i},i=1,\ldots ,N\right\}$ and $R=\left\{R_{i},i=1,\ldots ,N\right\}$ , where *i* is the cardiac beat counter and *N* is the series length, were separately converted into symbols via a uniform quantization procedure that covered the max–min range over *ξ* quantization bins (Porta et al., 2001; Cysarz et al., 2013). From the symbolic series ${HP}^{\xi }=\left\{{HP}_{i}^{\xi },i=1,\ldots ,N\right\}$ and $R^{\xi }=\left\{R_{i}^{\xi },i=1,\ldots ,N\right\}$ , we built the series of patterns of length *L*, ${HP}_{L}^{\xi }=\left\{{HP}_{i,L}^{\xi },i=L,\ldots ,N\right\}$ and $R_{L}^{\xi }=\left\{R_{i,L}^{\xi },i=L,\ldots ,N\right\}$ , where ${HP}_{i,L}^{\xi }=\left[\begin{array}{cc} \begin{array}{cc} {HP}_{i}^{\xi } & {HP}_{i-1}^{\xi } \end{array} & \begin{array}{cc} \ldots . & {HP}_{i-L+1}^{\xi } \end{array} \end{array}\right]$ and $R_{i,L}^{\xi }=\left[\begin{array}{cc} \begin{array}{cc} R_{i}^{\xi } & R_{i-1}^{\xi } \end{array} & \begin{array}{cc} \ldots . & R_{i-L+1}^{\xi } \end{array} \end{array}\right]$ are the patterns defined according to the technique of delay embedding. The parameters *ξ* = 6 and *L* = 3 were set according to Porta et al. (2001) to ensure the consistency of the estimates of the symbolic pattern rates over short series of HP and R with *N* = 256.

Each symbolic pattern was classified into one of four classes according to the variation between adjacent symbols (Porta et al., 2001): i) the no variation (0V) pattern when all the three symbols were equal; ii) the one variation (1V) pattern when two adjacent symbols were equal but the remaining one (i.e., the first or the third) was different; iii) the two like variation (2LV) pattern when the three symbols were different and showed a progressive increase, or decrease, from the first to the third one; iv) the two unlike variation (2UV) pattern when the two adjacent symbols were different with the second symbol of the pattern lower or higher than the first and the third one.

This approach allows the preservation of the amplitude features of the series according to the adopted coarse-graining procedure as well as the classification of patterns according to their temporal scale, given that the 0V pattern is the slowest one, because of its stable behavior, and the 2UV pattern is the fastest one, owing to its rapid changes. 1V and 2LV exhibited an intermediate range of time scales, with the 1V pattern slower than the 2LV one (Porta et al., 2001). The symbolization allowed the assessment of the autonomic control governing changes of HP (Guzzetti et al., 2005). The joint HP–R pattern was built by associating a pattern built over R, namely, $R_{i,L}^{\xi }$ , with a pattern built over HP *τ* lags ahead, namely, ${HP}_{i+\tau ,L}^{\xi }$ , where *τ* is an integer value, thus allowing the assessment of the association between HP and R with HP leading R for τ<0 , immediate HP–R association when τ=0 (i.e., within the current HP), and association between HP and R with HP lagging R for τ>0 .

We defined a joint coordinated (C) pattern associating one pattern built over HP and one pattern built over R when both belonged to the same family (Porta et al., 2015b). C patterns were labeled as 0V-0V, 1V-1V, 2LV-2LV, and 2UV-2UV. The percentage of C patterns (C%) over the total number of joint schemes (i.e*.*, $N-L-\left|\tau \right|+1$ ) was computed. C% was deemed to be an index of the overall association between the HP and R series regardless of the time scales of the HP–R dynamic interactions. C% ranged from 0 (i.e*.*, no coordinated behavior) to 100 (i.e*.*, all patterns are coordinated) (Porta et al., 2015b). The percentages of each of the four joint pattern classes were computed over the total number of C joint patterns for each *τ* value so that their sum was always 100%. These markers were denoted as 0V-0V%, 1V-1V%, 2LV-2LV%, and 2UV-2UV%. These four indexes were deemed to assess the association between the HP and R series at a specific time scale of interactions set by the type of joint pattern: HP–R dynamic interactions occurred at the slowest and fastest scales in the presence of 0V-0V and 2UV-2UV joint patterns, respectively, and at time scales intermediate between those described by the 0V-0V and 2UV-2UV joint schemes when the 1V-1V and 2L-2LV joint patterns were detected. Indexes ranged from 0 (i.e*.*, that specific scale did not contribute to the overall HP–R coordination) to 100 (i.e*.*, that specific scale fully explained the overall HP–R coordination) (Porta et al., 2015b).

In-phase and out-of-phase patterns, usually referred to as symmetric and diametric patterns (Wessel et al., 2009), were merged within the same class because classification did not account for the sign of the variations (Porta et al., 2001). In the range of breathing rates considered in the present study, the phase between heart rate and R volume was expected to be about 0 (Angelone and Coulter, 1964; Eckberg, 1983; Saul et al., 1989). Therefore, because we recorded HP, being in phase opposition with heart rate, and a nasal flow, being in quadrature with the R volume, the expected phase shift between HP and our R signal was π/2. This phase shift at a rate of 10 and 15 breaths·minute^−1^ was equivalent to latencies of 1.5 s and 1 s, respectively, namely, less than two beats at the cardiac frequencies of the present study in any experimental condition. Therefore, the selected interval for *τ* values was −2≤τ≤+2 .

## Simulations

To validate the ability of the LJSA approach to follow changes in the coupling strength, we simulated two stochastic processes that were interconnected unidirectionally or bidirectionally via a parameter modulating the coupling strength (Porta et al., 2023). The two processes $Y_{1}$ and $Y_{2}$ are defined as

$$\begin{array}{c} Y_{1,i}=2\rho_{1}\cdot \left[c_{1}\cdot Y_{2,i-1}+\left({1-c}_{1}\right)\cdot Y_{1,i-1}\right]\cdot \cos\varphi_{1}-\rho_{1}^{2}\cdot Y_{1,i-2}+W_{1,i},\\ Y_{2,i}=2\rho_{2}\cdot \left[c_{2}\cdot Y_{1,i-1}+\left({1-c}_{2}\right)\cdot Y_{2,i-1}\right]\cdot \cos\varphi_{2}-\rho_{2}^{2}\cdot Y_{2,i-2}+W_{2,i}, \end{array}$$ (1)

where $W_{1}$ and $W_{2}$ are Gaussian white noises with zero mean and variances assigned such that $Y_{1}$ and $Y_{2}$ exhibit unit variance. Three configurations are of value: i) full uncoupling between $Y_{1}$ and $Y_{2}$ with $c_{1}=c_{2}=0$ and $Y_{1}$ and $Y_{2}$ are two second-order autoregressive processes; ii) unidirectional interactions from $Y_{1}$ to $Y_{2}$ with $c_{1}=0$ and $c_{2}\neq 0$ ; iii) bidirectional interactions from $Y_{1}$ to $Y_{2}$ and vice versa with $c_{1}\neq 0$ and $c_{2}\neq 0$ . In the uncoupling condition, $Y_{1}$ and $Y_{2}$ were set to feature the same dominant rhythm according to $\rho_{1}=\rho_{2}=0.85$ with phases $\varphi_{1}=\varphi_{2}=\pm 3\pi /10$ corresponding to a dominant oscillation at the normalized frequency $f_{1}=f_{2}=0.15$ ${cycles\cdot sample}^{-1}$ , thus simulating the typical dynamics usually observed in HP and R series during SB and CB (i.e., 0.15 Hz with a mean HP equal to 1 s). In the unidirectional configuration from $Y_{1}$ to $Y_{2}$ , the coupling parameter $c_{2}$ was varied incrementally from 0 to 1.0 with 0.1 steps with $c_{1}=0$ . In the bidirectional configuration from $Y_{1}$ to $Y_{2}$ and *vice versa*, $c_{1}=c_{2}=c$ , and c was varied gradually from 0 to 1.0 with 0.1 steps.

## Experimental protocol and data analysis

### Experimental protocol

The estimate of CRC via LJSA was performed on a historical database designed to quantify cardiorespiratory interactions while varying the breathing rate (Porta et al., 2000b; Porta et al., 2012a). The protocol was in keeping with the Declaration of Helsinki and approved by the Local Ethical Review Board of L. Sacco Hospital, Milan, Italy (protocol code: 1999-3; date of approval: 1/2/1999). Written signed informed consent was obtained from all subjects. We enrolled 19 healthy subjects (age: 27–35 years, median = 31 years; 8 males, 11 females). The health status of subjects was verified via physical examination, evaluation of the historical personal records, blood pressure measurement with a sphygmomanometer, and standard assessment of the electrocardiographic trace by an expert cardiologist. During the experimental sessions, we acquired the electrocardiogram (ECG) from lead II via a bioamplifier (Marazza, Monza, Italy) and the R flow via a nasal thermistor (Marazza, Monza, Italy) at the sampling frequency of 300 Hz. The two signals were synchronized through a 12-bit analog-to-digital board (National Instruments, Austin, TX, United States) plugged into a personal computer. Recordings were made at rest in a supine position during SB, CB10, and CB15. The SB session always preceded CB recordings, while the order of the CB10 and CB15 sessions was randomized. The subjects were trained to follow a computer-based metronome that provided the pace for starting both the inspiratory and expiratory phases. The inspiratory-to-expiratory ratio was set to a traditional 1:2 (De Maria et al., 2021) via the metronome sounds. The indication provided by the metronome was reinforced through verbal commands by the experimenter. Sessions lasted 10 min, and subjects were not allowed to talk for the entire duration of the protocol.

### Beat-to-beat variability series extraction

The R-wave peaks were identified on the ECG through a threshold-based algorithm applied to the ECG first derivative. The *i*th HP value was then estimated as the time interval occurring between the *i*th and (*i* + 1)th R-wave peaks. The R signal was sampled at the *i*th R-wave peak. Sequences of 256 consecutive values were selected within each experimental condition, thus focusing on short-term cardiac control (Task Force of the European Society of Cardiology and the North American Society of Pacing and Electrophysiology, 1996). Detections of the R-wave peak were visually checked and manually corrected if necessary. The effect of any isolated arrhythmic beat was mitigated through linear interpolation using the two most adjacent HPs computed between sinus beats. The 5% limit of corrections was never reached. From the HP series, we computed the mean, labeled as $\mu_{HP}$ and expressed in ms.

### Frequency domain analysis

Power spectral density was estimated separately over the HP and R series via a parametric approach based on the identification of the coefficients of an autoregressive model (Baselli et al., 1997). The least squares problem was solved via the Levinson–Durbin recursion (Kay and Marple, 1981). The model order was optimized via the Akaike information criterion between 10 and 16 (Akaike, 1974). The traditional method based on the decomposition of the power spectral density according to the residue theorem was utilized to compute the power associated to each spectral component (Baselli et al., 1997). The high-frequency band (HF, 0.15–0.4 Hz) was considered for the computation of the RSA from the HP series and the assessment of the respiratory frequency (f_R_) from the R series. RSA was estimated by summing the power of all spectral components whose central frequency fell within the HF band, and this index was labeled as HF_HP_ and expressed in ms^2^. From the R series, we extracted the f_R_ as the central frequency of the dominant spectral component present in the HF band.

Power cross-spectral density was estimated over the HP and R series using a parametric approach based on the identification of the bivariate autoregressive model (Baselli et al., 1997). The least squares problem was solved via the Cholesky decomposition method (Porta et al., 2000a). The model order was fixed to 10. The power cross-spectral density was computed by taking the R series as the input and HP as the output. The phase $\varphi_{HP-R}\left(f\right)$ of the power cross-spectral density represented the phase shift from R to HP as a function of the frequency *f*, where negative values indicated that HP lagged R, and positive values indicated that HP led R. The phase was expressed in radians (rad) and ranged from −π to +π. The phase function was sampled at f_R_ $\left[\varphi_{HP-R}\left(f_{R}\right)\right]$ and converted into a time shift $\tau_{HP-R}\left(f_{R}\right)=\frac{0.5\cdot \varphi_{HP-R}\left(f_{R}\right)}{f_{R}\cdot \pi }$ expressed in s. $\tau_{HP-R}\left(f_{R}\right)$ is given in beats as well by dividing $\tau_{HP-R}\left(f_{R}\right)$ by $\mu_{HP}$ .

### Surrogate data approach

We utilized a surrogate data approach to test the null hypothesis of uncoupling between HP and R at a given time lag *τ* (H_0,τ_) and the null hypothesis of full uncoupling between HP and R regardless of the time lag (H_0_). We constructed sets of surrogate pairs preserving the distribution and power spectral density of the original series (Schreiber and Schmitz, 1996) but fully adherent with H_0,τ_ and H_0_ (Palus, 1997). We generated 100 realizations for each original pair of HP and R series in any experimental condition. The surrogate series were built to preserve the distribution and power spectral density of the original series, while phases were substituted with uniformly distributed random numbers ranging from 0 to 2π. Surrogate pairs were generated through an iteratively refined, amplitude-adjusted, Fourier transform-based procedure (Schreiber and Schmitz, 1996). The technique allowed the exact preservation of the original distribution, while the power spectral density was the best approximation of the initial power spectral density given 100 iterates. The use of two independent random phase sequences allowed the generation of fully uncoupled pairs (Palus, 1997). A fast Fourier transform procedure was applied to speed the construction of surrogates. The percentages of the C pattern families (i.e., 0V-0V%, 1V-1V%, 2LV-2LV%, and 2UV-2UV%) were computed over each set of surrogates, and the 95th percentile was extracted. At each time lag, if the percentage of the C pattern family computed over the original series was above the 95th percentile of the same index derived from surrogates and if this occurred for at least one of the 4°C pattern classes, the H_0,τ_ was rejected for the considered *τ*, and the alternative hypothesis, namely, the two series were significantly associated at the selected *τ*, was accepted. The *H*_*0*_ was rejected if H_0,r_ was rejected in correspondence with at least one of the considered time lags (i.e., τ = −2, τ = −1, τ = 0, τ = +1, or τ = +2), and the alternative hypothesis, namely, the two series were significantly associated regardless of time shift, was accepted. The percentage of H_0_ rejections was evaluated in any experimental condition (i.e., SB, CB10, and CB15).

### Statistical analysis

The normality of data was checked via the Shapiro–Wilk test. The one-way repeated measures analysis of variance versus control (Dunnett’s test for multiple comparisons), or the one-way Friedman repeated measures analysis of variance on ranks versus control (Dunn’s test for multiple comparisons), when appropriate, was used to separately evaluate the effect of breathing modality (i.e*.*, SB, CB10, and CB15) and the effect of time shift (i.e., *τ* = −2, *τ* = −1, *τ* = 0, *τ* = +1, and *τ* = +2). When assessing the impact of the breathing modality on LJSA markers, data were pooled regardless of the time lag. When assessing the impact of the time shift on LJSA markers, data were pooled regardless of the experimental condition. The two-way repeated measures analysis of variance versus control (Holm–Sidak test for multiple comparisons) was utilized to check the impact of the experimental condition within the same time lag and the effect of time lag within the same experimental condition. In the case of both the one-way and the two-way analyses, the control conditions were SB and *τ* = 0. The χ^2^ test (McNemar’s test) was applied to the proportion of subjects featuring the rejection of H_0_ to assess the impact of the CB versus SB. The level of significance of the test was lowered according to the number of comparisons (i.e., 2) to account for the multiple comparison issue. The same test was utilized to check the impact of CB versus SB within the same time lag and of the time lag versus τ = 0 within the same experimental condition on the proportions of subjects featuring the rejection of H_0,τ_. In this case, the level of significance was lowered by a factor accounting for all the considered comparisons. Statistical analysis was carried out using a commercial statistical program (SigmaPlot, v.14.0, Systat Software, Inc., Chicago, IL, United States). A *p* < 0.05 was always considered significant.

## Results

### Results over simulations

**FIGURE 1:**
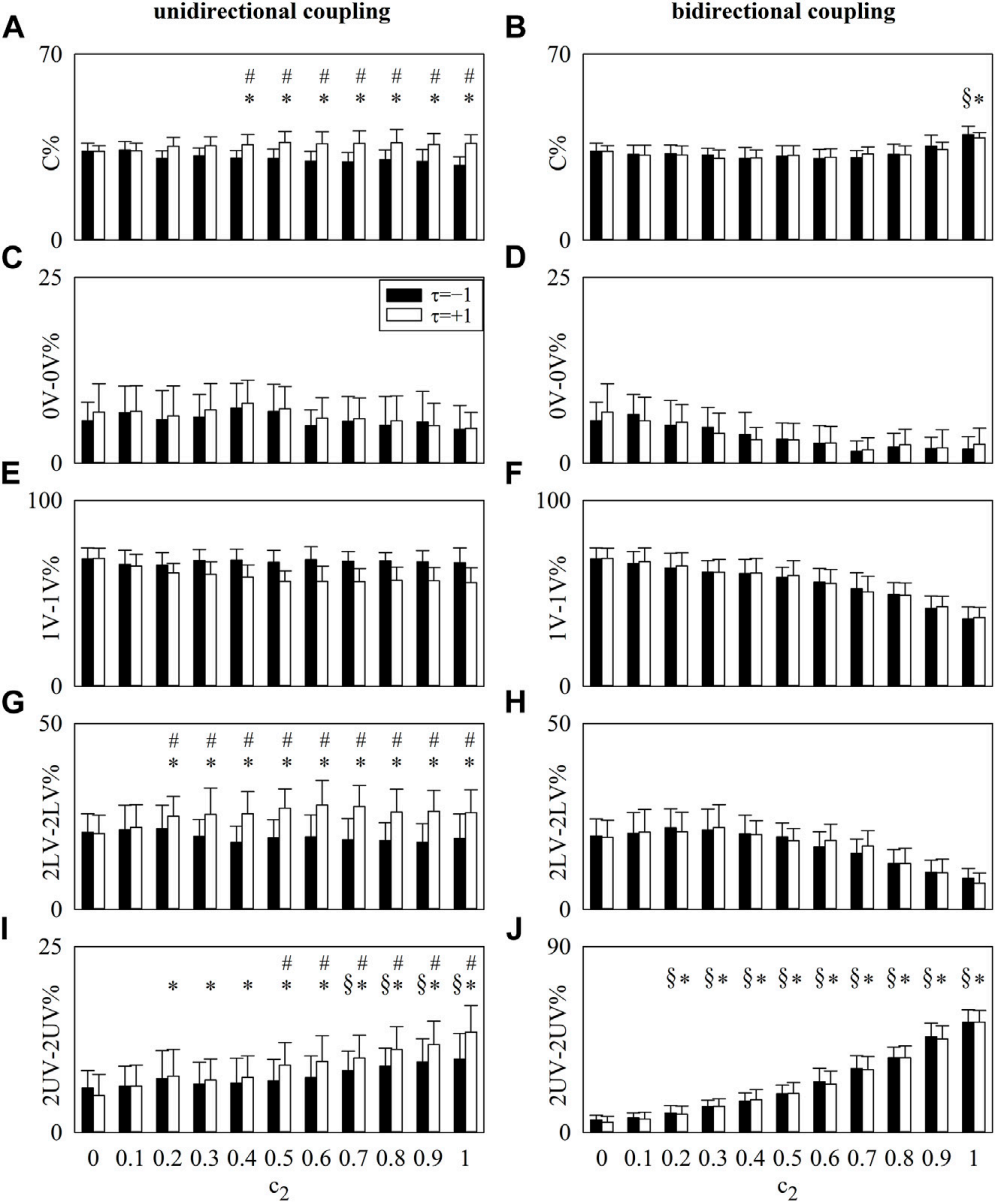
The vertical grouped error bar graphs show C% **(A,B)**, 0V-0V% **(C,D)**, 1V-1V% **(E,F)**, 2LV-2LV% **(G,H)**, and 2UV-2UV% **(I,J)** computed over simulated processes as a function of $c_{2}$ . Results were obtained in the case of unidirectional interactions with $c_{1}=0$ **(A,C,E,G,I)** and bidirectional interactions with $c_{1}=c_{2}$ **(B,D,F,H,J)** from 20 realizations of $Y_{1}$ and $Y_{2}$ . Results were reported for τ=−1 (black bars) and τ=+1 (white bars). At $c_{2}=0$ , $Y_{1}$ and $Y_{2}$ were uncoupled. The symbols § and * indicate a significant difference with *p* < 0.05 versus $c_{2}=0$ within the same time shift, being τ=−1 and τ=+1 , respectively. The symbol # indicates a significant difference with *p* < 0.05 between the time lags within the same value of $c_{2}$ . Symbols indicating *p* < 0.05 were reported solely when the null hypothesis of uncoupling between $Y_{1}$ and $Y_{2}$ was rejected (i.e., the value of the markers was significantly above the one found at $c_{2}=0$ ). Data are reported as mean plus standard deviation.

**FIGURE 2:**
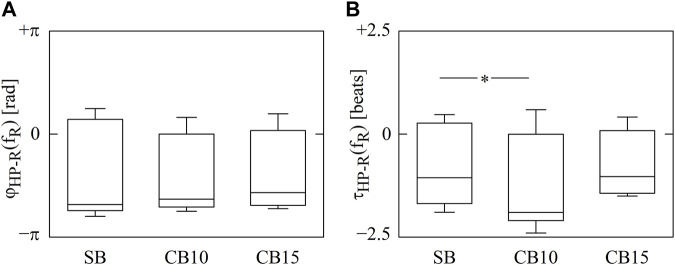
The vertical box-and-whisker plots show $\varphi_{HP-R}\left(f_{R}\right)$ **(A)** and $\tau_{HP-R}\left(f_{R}\right)$ **(B)** as a function of the experimental condition (i.e., SB, CB10, and CB15). The height of the box represents the distance between the first and third quartiles, with the median marked as a horizontal segment, and the whiskers denote the 5th and 95th percentiles. The symbol * indicates *p* < 0.05 versus SB.

The vertical grouped error bar graphs of Figure 1 summarize the results of simulations of unidirectionally coupled (Figures 1A, C, E, G, I) and bidirectionally coupled (Figures 1B, D, F, H, J) stochastic processes. Results are shown for τ=−1 (black bars) and τ=+1 (white bars), given that $Y_{1}$ acts on $Y_{2}$ with τ=+1 in the simulations of unidirectionally coupled processes and lagged interactions in the simulations of bidirectionally coupled processes occurred with τ=+1 from $Y_{1}$ to $Y_{2}$ and *vice versa*. Results are provided for C% (Figures 1A, B), 0V-0V% (Figures 1C,D), 1V-1V% (Figures 1E, F), 2LV-2LV% (Figures 1G, H), and 2UV-2UV% (Figures 1I, J) as a function of the parameter $c_{2}$ regulating the coupling strength between the processes. Both simulations are designed in such a way that $c_{2}=0$ corresponds to the situation of uncoupling between realizations of stochastic processes featuring a dominant oscillation at the same frequency compatible with the breathing rate of 0.15 Hz with an HP = 1 s, and the coupling strength grows with $c_{2}$ . Results were obtained from 20 pairs of realizations.

In the case of unidirectionally coupled processes, C%, 2LV-2LV%, and 2UV-2UV% computed at τ=+1 were larger than the level set by the uncoupling condition (i.e., at $c_{2}=0$ ) when $c_{2}\geq 0.4$ , $c_{2}\geq 0.2,$ and $c_{2}\geq 0.2$ , respectively, and these indexes were larger than the value obtained at τ=−1 when $c_{2}\geq 0.4$ , $c_{2}\geq 0.2$ , and $c_{2}\geq 0.5$ . Remarkably, 2UV-2UV% increased gradually with $c_{2}$ . 0V-0V% and 1V-1V% remained less than the value set by the uncoupling condition (i.e., at $c_{2}=0$ ), regardless of the time shift, because the link between the two processes occurred at fast time scales. 2UV-2UV% at τ=−1 was larger than the value set by the uncoupled realizations as well, but it remained less than its value at τ=+1 , and the rate of the increase was less steep than that at τ=+1 .

In the case of bidirectionally coupled processes, C% and 2UV-2UV% computed at both τ=±1 were larger than the level set by the uncoupling condition (i.e., at $c_{2}=0$ ) when $c_{2}\geq 1.0$ and $c_{2}\geq 0.2$ , respectively. C% and 2UV-2UV% were alike when computed with τ=±1 . As in the case of the unidirectionally coupled processes, 2UV-2UV% increased gradually with $c_{2}$ . 0V-0V%, 1V-1V%, and 2LV-2LV% remained less than the value set by the uncoupling condition (i.e., at $c_{2}=0$ ), regardless of the time shift.

### Impact of CB on time, spectral and cross-spectral markers

The results of the time and frequency domain analysis of the HP variability and R signal are reported in Table 1. The μ_HP_ and HF_HP_ powers were similar across experimental conditions. f_R_ did not change during CB15 compared to SB, while it was significantly slower during CB10.

**TABLE 1:** Time and frequency domain markers derived from the HP variability and R signal.

Index	SB	CB10	CB15
μ_HP_ [ms]	1,010 ± 168	989 ± 157	1,023 ± 162
HF_HP_ [ms^2^]	1,390 ± 1,631	2,800 ± 2,986	1855 ± 1873
f_R_ [breaths·minute^−1^]	14.7 ± 1.7	12.1 ± 2.5§	14.4 ± 1.2

SB, spontaneous breathing; CB10, controlled breathing at 10 breaths·minute^−1^; CB15, controlled breathing at 15 breaths·minute^−1^; HP, heart period; R, respiration; μ_HP_, mean HP; HF_HP_, HP power in the HF band; f_R_, respiratory rate. All values are expressed as mean ± standard deviation. The symbol § indicates *p* < 0.05 vs. SB.

The vertical box-and-whisker plots of Figure 2 show cross-spectral markers, namely, $\varphi_{HP-R}\left(f_{R}\right)$ (Figure 2A) and $\tau_{HP-R}\left(f_{R}\right)$ (Figure 2B), as a function of the experimental condition (i.e., SB, CB10, and CB15). The height of the box represents the distance between the first and third quartiles, with the median marked as a horizontal segment, and the whiskers denote the 5th and 95th percentiles. The phase is expressed in rad (Figure 2A) and converted into a delay/advancement expressed in s in Figure 2B. Significance was tested against SB. Phases were more likely to be negative, thus indicating that, most frequently, HP lagged R. The median values were −2.15, −1.98, and −1.78 rad during SB, CB10, and CB15, respectively (Figure 2A). After converting phase values into lags (Figure 2B), the delay of HP to R was more pronounced during CB10 than SB and CB15, with median values of −0.71, −1.31, and −0.69 beats during SB, CB10, and CB15, respectively, and individual values being more negative than −2 beats in 5%, 32%, and 0% and never more positive than +2 beats during SB, CB10, and CB15, respectively.

### Results of the surrogate data test

**FIGURE 3:**
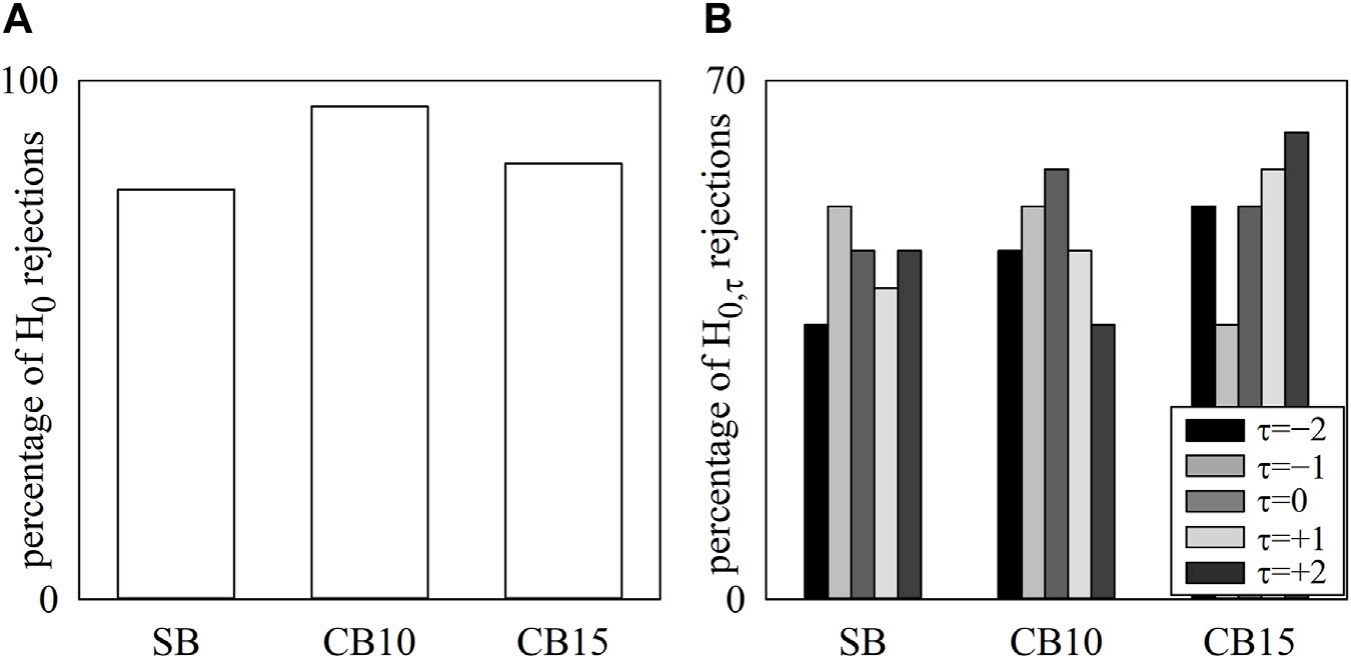
The simple bar graphs show the percentage of H_0_ rejections as a function of the experimental condition (i.e., SB, CB10, and CB15) **(A)**, and the grouped bar graphs show the percentage of H_0,τ_ rejections as a function of the experimental condition with the time lag *τ* coded according to the filling color of the bar from light gray to black **(B)**. No significant differences were detected across experimental conditions and lags.

The simple bar graphs of Figure 3A show the percentage of H_0_ rejections as a function of the experimental condition, while the grouped bar graph of Figure 3B summarizes the percentage of H_0,τ_ rejections as a function of the experimental condition at any time lag τ represented with bars of different filling color from light gray to black. Regardless of the experimental condition, HP and R series were found to be significantly coupled (above 79%), and the percentage of H_0_ rejections peaked at 95% and 84% during CB10 and CB15, respectively (Figure 3A). Remarkably, the percentage of H_0,τ_ rejections did not depend on either the time lag or experimental condition, with values ranging from 37% to 63% (Figure 3B).

### Impact of CB and time lag on LJSA markers

**FIGURE 4:**
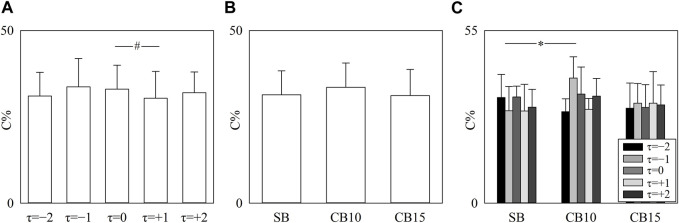
The simple error bar graphs show C% as a function of the time lag (i.e., to *τ* = −2, *τ* = −1, *τ* = 0, *τ* = +1, and *τ* = +2) when data are pooled regardless of the experimental condition **(A)** and C% as a function of the experimental condition (i.e., SB, CB10, and CB15) when data are pooled regardless of the time lag **(B)**. The grouped error bar graphs show C% as a function of the experimental condition with *τ* coded according to the filling color of the bar from light gray to black **(C)**. The symbol # indicates a significant between-time lag difference versus *τ* = 0 with *p* < 0.05. Data are reported as mean plus standard deviation.

The simple error bar graph of Figure 4A shows C% as a function of the time lag after pooling all the data regardless of the experimental condition, while the simple error bar graph of Figure 4B shows C% as a function of the experimental condition after pooling all the data regardless of the time shift. The grouped error bar graph of Figure 4C summarizes C% as a function of the experimental condition when the time shift is represented by different filling colors of the bars from light gray to black. Significance was tested against τ = 0 and SB. C% decreased at τ = +1 compared to τ = 0 (Figure 4A), did not vary with experimental condition (Figure 4B), and increased at τ = −1 during CB10 compared to SB (Figure 4C).

**FIGURE 5:**
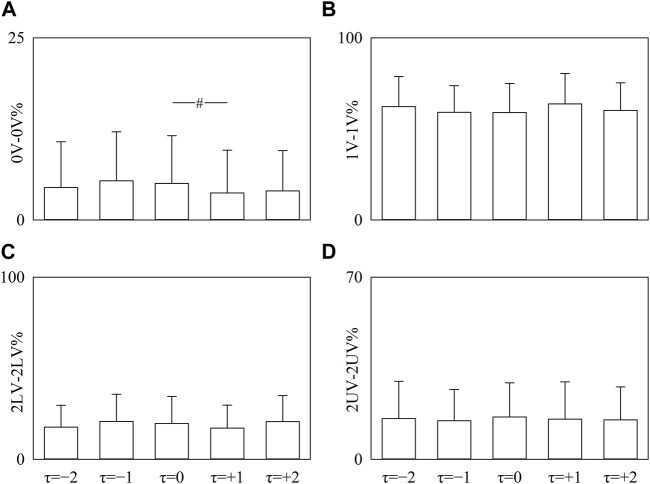
The vertical grouped error bar graphs show 0V-0 V% **(A)**, 1V-1V% **(B)**, 2LV-2LV% **(C)**, and 2UV-2UV% **(D)** as a function of the time lag (i.e., to *τ* = −2, *τ* = −1, *τ* = 0, *τ* = +1, and *τ* = +2). Data are pooled regardless of the experimental condition. The symbol # indicates a significant between-time lag difference versus *τ* = 0 with *p* < 0.05. Data are reported as mean plus standard deviation.

The simple error bar graphs of Figure 5 report 0V-0V% (Figure 5A), 1V-1V% (Figure 5B), 2LV-2LV% (Figure 5C), and 2UV-2UV% (Figure 5D) as a function of the time shift when data are pooled regardless of the experimental condition. Significance was tested against τ = 0. 0V-0V% decreased at τ = +1 (Figure 5A), while 1V-1V%, 2LV-2LV%, and 2UV-2UV% did not vary with τ with respect to τ = 0 (Figures 5B–D).

**FIGURE 6:**
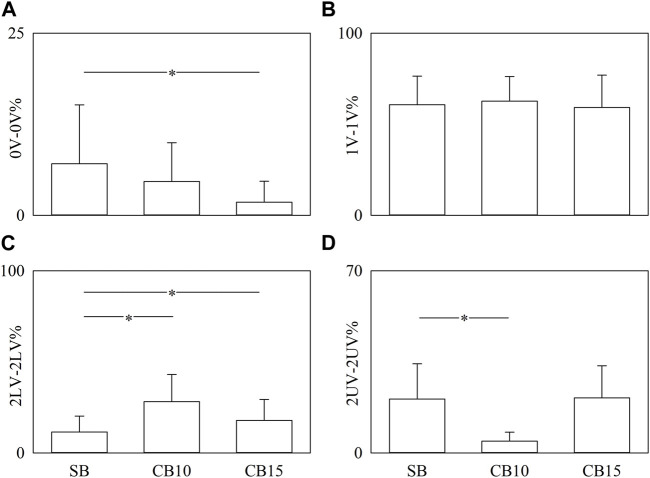
The vertical grouped error bar graphs show 0V-0V% **(A)**, 1V-1V% **(B)**, 2LV-2LV% **(C)**, and 2UV-2UV% **(D)** as a function of experimental condition (i.e., SB, CB10, and CB15). Data are pooled regardless of the time lag between HP and R series. The symbol * indicates a significant between-experimental condition difference versus SB with *p* < 0.05. Data are reported as mean plus standard deviation.

Figure 6 has the same structure as Figure 5, but the percentages of pattern classes are given as a function of the experimental condition when data are pooled regardless of the time lag. Significance was tested against SB. 0V-0V% significantly decreased solely during CB15 (Figure 6A), while the decrease of 2UV-2UV% was evident solely during CB10 (Figure 6D). 2LV-2LV% increased both during CB10 and CB15 (Figure 6C). CB did not affect 1V-1V% (Figure 6B).

**FIGURE 7:**
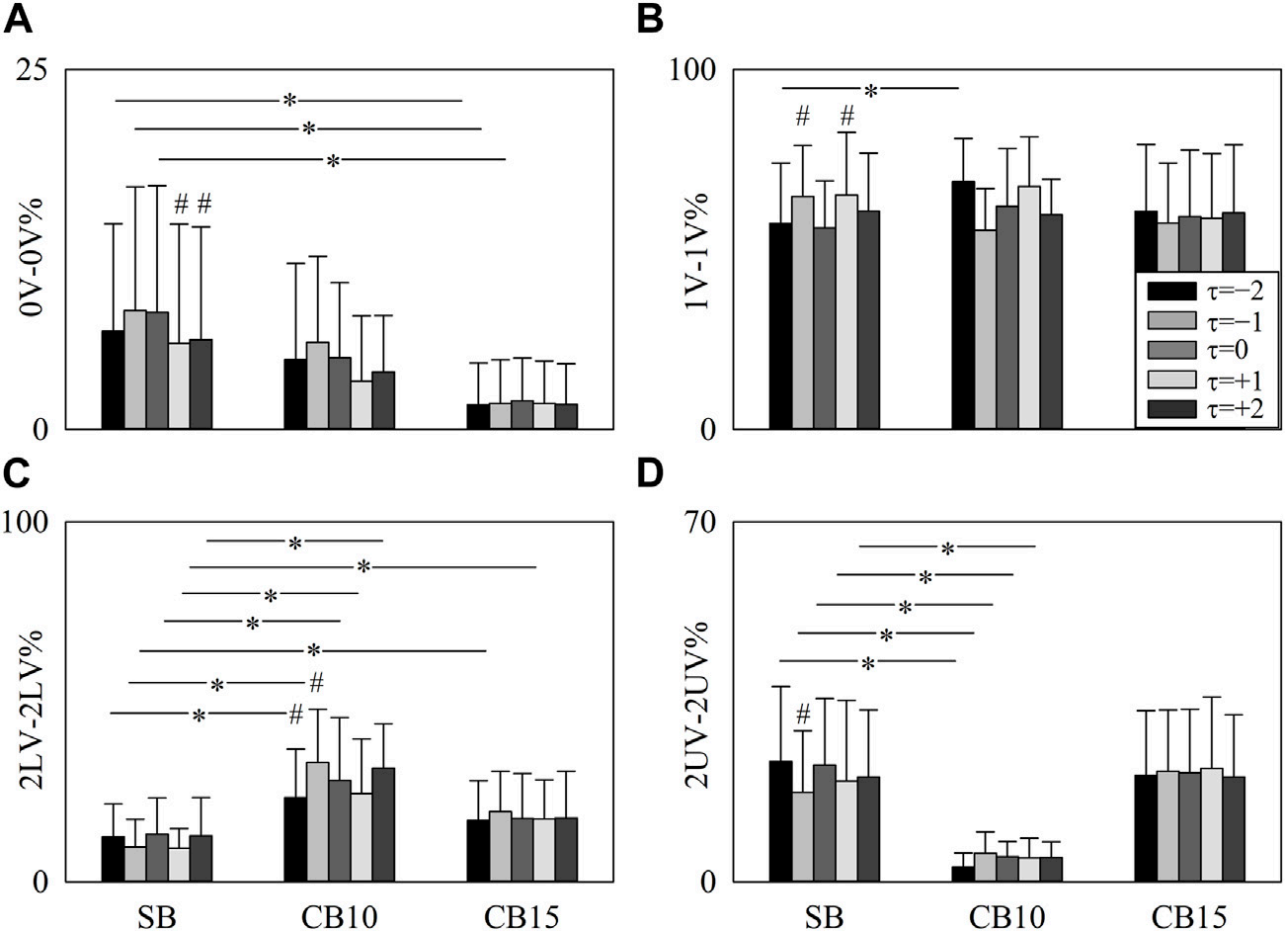
The vertical grouped error bar graphs show 0V-0V% **(A)**, 1V-1V% **(B)**, 2LV-2LV% **(C)**, and 2UV-2UV% **(D)** as a function of experimental condition (i.e., SB, CB10, and CB15). Data relevant to *τ* = −2, *τ* = −1, *τ* = 0, *τ* = +1, and *τ* = +2 are coded according to the filling color of the bar from light gray to black. The symbol * indicates a significant between-experimental condition difference versus SB with *p* < 0.05 within the same time shift (i.e., to *τ* = −2, *τ* = −1, *τ* = 0, *τ* = +1, or *τ* = +2). The symbol # indicates a significant between-time lag difference with *p* < 0.05 within the same experimental condition (i.e., SB, CB10, or CB15). Data are reported as mean plus standard deviation.

The grouped error bar graphs of Figure 7 summarize 0V-0V% (Figure 7A), 1V-1V% (Figure 7B), 2LV-2LV% (Figure 7C), and 2UV-2UV% (Figure 7D) as a function of the experimental condition with the time shift τ represented as the filling color of the bar from light gray to black. Significance was tested against SB, and τ = 0. 0V-0V% decreased during CB15 compared to SB when τ = −2, τ = −1, and τ = 0 (Figure 7A). During SB, 0V-0V% was smaller at τ = +1 and τ = +2 compared to τ = 0 (Figure 7A). At τ = −2, 1V-1V% increased during CB10 compared to SB, and during SB, 1V-1V% was higher at τ = −1 and τ = +1 compared to τ = 0 (Figure 7B). At any time lag, 2LV-2LV% increased during CB10 compared to SB (Figure 7C). The increase of 2LV-2LV% was evident even during CB15 but solely at τ = −1 and τ = +1 (Figure 7C). During CB10, 2LV-2LV% increased at τ = −1 and decreased at τ = −2 compared to τ = 0 (Figure 7C). At any time lag, 2UV-2UV% declined during CB10 compared to SB (Figure 7D). During SB, 2UV-2UV% decreased at τ = −1 compared to τ = 0 (Figure 7D).

## Discussion

The main findings of this study can be summarized as follows: ii) LJSA was useful to quantify the CRC strength while testing different temporal schemes and time scales of the HP–R interactions; ii) the test performed over surrogate data proved that HP and R were significantly coupled in any experimental condition and coupling did not occur more likely at a specific time lag; iii) CB10 reduced the CRC strength at fast time scales and increased it at intermediate time scales, thus leaving the overall CRC strength unvaried; iv) despite exhibiting similar R rates and RSA values, SB and CB15 induced different cardiorespiratory interactions; v) no dominant time-shifted coordinated pattern was observed with relevant contributions of HP patterns either leading or lagging R.

### Characterizing CRC through LJSA

We exploited LJSA (Baumert et al., 2002; Wessel et al., 2009; Kabir et al., 2011; Porta et al., 2015a; Porta et al., 2015b) to typify CRC from spontaneous fluctuations of HP and R according to the implementation proposed in Porta et al. (2015b). The characterization is grounded on the assessment of the probability of joint patterns, expressed as a percentage, describing coordinated behaviors between HP changes and R (i.e., C%). The C patterns were classified according to the frequency content of the features comprising the joint behavior from the slowest (i.e., 0V-0V) to the most rapid (i.e., 2UV-2UV) passing through intermediate levels of rapidity (i.e., 1V-1V and 2LV-2LV), thus allowing the assessment of the contribution of time scales to the total coordination. The joint pattern is formed by imposing a time shift between the symbolic patterns, built separately over the two series and combined to form the joint pattern in such a way as to test different temporal schemes of cardiorespiratory interactions (Baumert et al., 2002; Wessel et al., 2009; Kabir et al., 2011): negative time shifts imply that HP leads R, no time shift implies HP–R immediate interaction, namely, associations between HP and R within the current HP, and positive time shifts imply that HP lags behind R. According to the definition of patterns built separately over the two series, both in-phase and out-of-phase schemes are present within the same LJSA class (Wessel et al., 2009; Suhrbier et al., 2010). The selected approach has three potential strengths: i) being a model-free approach, it can describe nonlinear interactions between HP variability and R (Baumert et al., 2002; Wessel et al., 2009; Kabir et al., 2011; Porta et al., 2015a; Porta et al., 2015b); ii) different temporal schemes can be easily introduced by setting the delay/advancement between patterns built separately over HP variability and R (Wessel et al., 2009; Suhrbier et al., 2010; Kabir et al., 2011; Baumert et al., 2015); iii) different time scales of interactions can be explored according to the definition of the joint patterns (Porta et al., 2015b; Bari et al., 2016; Porta et al., 2016).

The range of lags explored in the present analysis was limited according to previous studies (Angelone and Coulter, 1964; Eckberg, 1983; Saul et al., 1989). Angelone and Coulter (1964) suggested that heart rate and thoracic movements are in-phase at *f*_*R*_ = 10 breaths·minute^−1^. Eckberg (1983) extended this observation by observing that the onset of HP shortening occurs progressively earlier during inspiration while slowing R such that it precedes the onset of inspiration at *f*_*R*_ = 8 breaths·minute^−1^. Through a broad-band respiratory approach and cross-spectral analysis between heart rate and R volume, Saul et al. (1989) found that in the range of R frequencies between 0.15 and 0.25 Hz, heart rate and R volume interact with minimal lead (i.e., about zero phase), while robust phase leads are observed below 0.15 Hz. Therefore, because we recorded HP, being in phase opposition with heart rate, and an R flow signal, being in quadrature with the R volume, our two series are expected to be in quadrature. Cross-spectral analysis confirmed that the absolute value of the median phase at the R rate was smaller than π/2 in all the experimental conditions, and the values of lags expressed in samples are between −2 and +2 beats. Although the phase values are mostly negative, thus indicating that HP lagged R, the variability of phase values suggested the necessity of exploring even the positive phase, compatible with the observation that HP changes might precede R variations. The relevance of this exploration is supported by previous studies stressing the presence of a pathway from the heart to the respiratory system, even in healthy subjects (Tzeng et al., 2003; Larsen et al., 2010; Friedman et al., 2012). The relevance of this pathway was confirmed by modeling approaches as well (Yana et al., 1993; Perrott and Cohen, 1996; Porta et al., 2013b). The procedure adopted in the present study based on cross-spectral analysis allows the optimization of the range of lags compared to studies where a much wider interval of lags was considered (Galletly and Larsen, 1997), thus limiting the number of statistical comparisons and increasing the statistical power of the analysis. In addition, LJSA could provide a unique possibility of monitoring the evolution of the CRC strength by simultaneously accounting for nonlinearity, delays/advancements, and time scales of the HP–R dynamic interactions. These characteristics result from the model-free nature of the method, the introduction of the time shift between HP and R patterns, and the types of joint schemes combining HP and R patterns that interact at different frequencies.

### Coupling strength and LJSA

Simulations support the ability of LJSA to follow variations of the coupling strength. Indeed, in situations of unidirectional or bidirectional coupling set between processes exhibiting a dominant and fast component, markers describing the association between realizations occurring at the fastest time scale were able to follow the progressive increase of the coupling strength. It is worth noting that an unspecific marker of association such as C% is much less powerful than a very specific index such as 2UV-2UV%, thus corroborating the relevance of following a specific class of joint pattern selected according to whether coupling could occur at faster or slower time scales. However, given the shortness of the considered patterns combined to form the joint scheme, the time scales of interactions remain roughly defined, but this characterization is sufficient to separate different classes of interactions. Differences between markers computed over different joint pattern classes support the relationship of the indexes with the temporal scheme of interactions and their ability to separate leading interactions from lagging ones (*e.g.*, in the simulation of unidirectionally coupled processes). Because 0V-0V, 1V-1V, 2LV-2LV, and 2UV-2UV patterns belong to the class of C pattern, an increased percentage of one or more classes corresponds to a decrease of at least one of the others. This situation is evident in the simulation of bidirectionally coupled processes when 2UV-2UV% increased above the level set by the uncoupling situation and gradually increased with the coupling strength, while all the other classes became insignificant (i.e., less than the level of uncoupling). This effect might be particularly useful when coupling occurs at a dominant time scale, like in CRC, but it might be particularly limiting in situations where the association occurs simultaneously at different time scales.

### CB affects CRC even in the absence of RSA modification

The invariable value of C% with the experimental condition might suggest that CRC strength did not vary during CB. This result disagrees with studies that suggested that CRC strength increases while slowing R (Porta et al., 2000b; Porta et al., 2023). However, when the analysis was made more specific by accounting for the time scales of the interactions governing CRC, we observed that 2LV-2LV% increased and 2UV-2UV% decreased during CB10, thus suggesting that slowing the f_R_ diminishes the coordination between HP and R at the fastest time scale, but it improves it at intermediate time scale. It has been proven that CB at the same f_R_ as the SB did not alter RSA (Patwardhan et al., 1995; Pinna et al., 2006). The present study confirms this finding. However, SB and CB15 could not be considered indistinguishable. Indeed, 0V-0V% decreased and 2LV-2LV% increased during CB15 compared to SB as a likely result of the smaller impact of slow fluctuations on the HP series and a greater regularization of the HP fluctuations occurring at the f_R_ (Porta et al., 2000a). Because the practice of CB is widely utilized to standardize the impact of f_R_ on HP variability analysis (Patwardhan et al., 1995; Pinna et al., 2006), the present finding suggests that CB might have the wanted effects over some linear univariate parameters, such as RSA, but it might be ineffective, and even introduce additional confounding influences, over nonlinear bivariate markers.

### No predominant HP–R temporal scheme is observed

As previously observed in Kabir et al. (2011) and Baumert et al. (2015), introducing a lag between HP and R might lead to observing higher values of CRC strength. However, the procedure adopted to maximize the value of CRC strength should also take into consideration that HP might lead R, in addition to lagged interactions of HP to R (Wessel et al., 2009; Suhrbier et al., 2010). Indeed, the pathway from HP to R is plausible because a constant latency between the inspiratory onset and the last heartbeat preceding it was found (Tzeng et al., 2003; Larsen et al., 2010; Friedman et al., 2012), even at physiological breathing rates (Eckberg, 1983; Saul et al., 1989). It is worth noting that the values of lag to be explored should be carefully chosen according to the type of the recorded signals, here HP and R flow, and with the help of specific analyses, here cross-spectral analysis, limiting the interval of values to be tested. The present study suggests that there is no dominant temporal scheme in the HP–R dynamic interactions, given that LJSA markers did not show a clear behavior at a specific time lag. Conversely, the presence of some peaks at both positive and negative time lags suggests the possible presence of closed-loop interactions, given that the open-loop condition should be characterized by the dominant presence of an assigned temporal scheme as suggested by simulations of unidirectionally coupled processes. Therefore, contrary to the warning raised in Larsen et al. (2010), our data do not support the statement that the CB could disrupt the anticipatory action of HP on R and highlight the importance of considering closed-loop models when describing the dynamic relationship between HP and R (Larsen et al., 2010; Porta et al., 2013b) instead of the more traditional approaches that exclusively model the action from R to HP (Baselli et al., 1994; Triedman et al., 1995; Porta et al., 2000b; Chen and Mukkamala, 2008; Porta et al., 2012a; Porta et al., 2012b; Porta et al., 2013a). While the pathway from R to HP is usually considered to be the result of the activity of the respiratory centers in the brain stem modulating vagal and sympathetic motoneuron firing and, in turn, sinus node activity (Gilbey et al., 1984; Eckberg, 2003; Skytioti and Elstad, 2022), fewer hypotheses have been advanced regarding potential physiological mechanisms underpinning interactions from the heart to the respiratory system. It has been proposed that HP changes at the f_R_ precede modifications of the R signal due to the rapidity of vagal cardiac control compared to the slowness of modifications of the R volume (Yana et al., 1993; Triedman et al., 1995; Perrott and Cohen, 1996; Porta et al., 2013b). Less trivially, it has been suggested that modifications of the baroreceptor activity during systole occurring in the late expiration could contribute to starting the inspiratory phase at a preferred latency (Galletly and Larsen, 1999; Dick and Morris, 2004; Dick and Morris, 2004) and modulating the CRC strength (Abreu et al., 2020).

## Conclusion

This study evaluates the effect of SB and CB on CRC strength in healthy subjects using LJSA over HP variability and R signal. LJSA accounts for temporal schemes with either HP leading or lagging R. Results show that CB has an impact on CRC strength not only when the breathing rate is slower than that of SB but also when the rates are similar. In addition, the study proves that in healthy subjects, no dominant temporal scheme governing HP–R dynamic interactions is present with relevant contributions of HP patterns either leading or lagging R. This result is compatible with closed-loop dynamic interactions between the heart and the respiratory system. More experiments are needed to further elucidate the physiological meaning and mechanisms behind the observed closed-loop interactions and whether results might depend on the strategy adopted for LJSA computation, including symbolization and pattern construction procedures. In addition, future studies should compare the information derived from measures of association, like those proposed in the present study, with indexes more specifically designed to assess the directionality of coupling and causality (Bahraminasab et al., 2008; Staniek and Lehnertz, 2008; Chicarro and Andrzejak, 2009; Wessel et al., 2009; Suhrbier et al., 2010; Li et al., 2011; Martini et al., 2011; Dicken and Lehnertz, 2014).

## Data Availability

The dataset is relevant to the ESGCO 2022 challenge, “Characterizing cardiorespiratory interactions from spontaneous fluctuations of heart period and respiratory flow during controlled breathings,” and it can be downloaded without any restriction at https://www.esgco.org/challenges.

## References

[B1] Abreu R. M. Cairo B. Porta A. (2023). On the significance of estimating cardiorespiratory coupling strength in sports medicine. Front. Netw. Physiol. 2, 60. 10.3389/fnetp.2022.1114733 PMC10013023 36926078

[B2] Abreu R. M. Catai A. M. Cairo B. Rehder-Santos P. De Maria B. Vaini E. (2019). “Assessment of the coupling strength of cardiovascular control via joint symbolic analysis during postural challenge in recreational athletes,” in Proceedings of the Annual International Conference of the IEEE Engineering in Medicine and Biology Society, Berlin, Germany, 23-27 July 2019 (IEEE), 2011–2014. 10.1109/EMBC.2019.8857213 31946295

[B3] Abreu R. M. Catai A. M. Cairo B. Rehder-Santos P. Silva C. D. Favari Signini E. (2020). A transfer entropy approach for the assessment of the impact of inspiratory muscle training on the cardiorespiratory coupling of amateur cyclists. Front. Physiol. 11, 134. 10.3389/fphys.2020.00134 32158402 PMC7052290

[B4] Akaike H. (1974). A new look at the statistical model identification. IEEE Trans. Autom. Control 19, 716–723. 10.1109/tac.1974.1100705

[B5] Angelone A. Coulter N. A. (1964). Respiratory sinus arrhythmina: A frequency dependent phenomenon. J. Appl. Physiol. 19, 479–482. 10.1152/jappl.1964.19.3.479 14173545

[B6] Bahraminasab A. Ghasemi F. Stefanovska A. McClintock P. V. E. Kantz H. (2008). Direction of coupling from phases of interacting oscillators: A permutation information approach. Phys. Rev. Lett. 100, 084101. 10.1103/PhysRevLett.100.084101 18352623

[B7] Bari V. Marchi A. De Maria B. Rossato G. Nollo G. Faes L. (2016). Nonlinear effects of respiration on the crosstalk between cardiovascular and cerebrovascular control systems. Phil. Trans. R. Soc. A 374, 20150179. 10.1098/rsta.2015.0179 27044988

[B8] Bartsch R. P. Schumann A. Y. Kantelhardt J. W. Penzel T. Ivanov P. Ch. (2012). Phase transitions in physiologic coupling. Proc. Natl. Acad. Sci. 109, 10181–10186. 10.1073/pnas.1204568109 22691492 PMC3387128

[B9] Baselli G. Cerutti S. Badilini F. Biancardi L. Porta A. Pagani M. (1994). Model for the assessment of heart period and arterial pressure variability interactions and of respiration influences. Med. Biol. Eng. Comput. 32, 143–152. 10.1007/BF02518911 8022210

[B10] Baselli G. Porta A. Rimoldi O. Pagani M. Cerutti S. (1997). Spectral decomposition in multichannel recordings based on multi-variate parametric identification. IEEE Trans. Biomed. Eng. 44, 1092–1101. 10.1109/10.641336 9353988

[B11] Baumert M. Walther T. Hopfe J. Stepan H. Faber R. Voss A. (2002). Joint symbolic dynamic analysis of beat-to-beat interactions of heart rate and systolic blood pressure in normal pregnancy. Med. Biol. Eng. Comput. 40, 241–245. 10.1007/BF02348131 12043807

[B12] Baumert M. Javorka M. Kabir M. (2015). Joint symbolic dynamics for the assessment of cardiovascular and cardiorespiratory interactions. Phil. Tran. R. Soc. A 373, 20140097. 10.1098/rsta.2014.0097 PMC4281868 25548272

[B13] Brown T. E. Beightol L. A. Koh J. Eckberg D. L. (1993). Important influence of respiration on human R-R interval power spectra is largely ignored. J. Appl. Physiol. 75, 2310–2317. 10.1152/jappl.1993.75.5.2310 8307890

[B14] Cairo B. Abreu R. M. Bari V. Gelpi F. De Maria B. Rehder-Santos P. (2021). Optimizing phase variability threshold for automated synchrogram analysis of cardiorespiratory interactions in amateur cyclists. Phil. Trans. R. Soc. A 379, 20200251. 10.1098/rsta.2020.0251 34689616

[B15] Cairo B. Bari V. Gelpi F. De Maria B. Porta A. (2022). “ESGCO 2022 challenge: Joint symbolic analysis characterizes cardiorespiratory coupling in healthy subjects,” in 12th Conference of the European Study Group on Cardiovascular Oscillations (ESGCO 2022), Vysoké Tatry, Štrbské Pleso, Slovakia, 09-12 October 2022 (IEEE). 10.1109/ESGCO55423.2022.9931349

[B16] Chen X. Mukkamala R. (2008). Selective quantification of the cardiac sympathetic and parasympathetic nervous systems by multisignal analysis of cardiorespiratory variability. Am. J. Physiol. 294, H362–H371. 10.1152/ajpheart.01061.2007 17993596

[B17] Chicharro D. Andrzejak R. G. (2009). Reliable detection of directional couplings using rank statistics. Phys. Rev. E 80, 026217. 10.1103/PhysRevE.80.026217 19792241

[B18] Cysarz D. Porta A. Montano N. van Leeuwen P. Kurths J. Wessel N. (2013). Quantifying heart rate dynamics using different approaches of symbolic dynamics. Eur. Phys. J. Spec. Top. 222, 487–500. 10.1140/epjst/e2013-01854-7 24110868

[B19] De Maria B. Dalla Vecchia L. A. Maestri R. Pinna G. D. Parati M. Perego F. (2021). Lack of association between heart period variability asymmetry and respiratory sinus arrhythmia in healthy and chronic heart failure individuals. PLoS ONE 16, e0247145. 10.1371/journal.pone.0247145 33592077 PMC7886158

[B20] Dick T. E. Morris K. F. (2004). Quantitative analysis of cardiovascular modulation in respiratory neural activity. J. Physiol. 556, 959–970. 10.1113/jphysiol.2003.060418 14978205 PMC1664997

[B21] Dick T. E. Shannon R. Lindsey B. G. Nuding S. C. Segers L. S. Baekey D. M. (2005). Arterial pulse modulated activity is expressed in respiratory neural output. J. Appl. Physiol. 99, 691–698. 10.1152/japplphysiol.01124.2004 15761086

[B22] Dickten H. Lehnertz K. (2014). Identifying delayed directional couplings with symbolic transfer entropy. Phys. Rev. E 90, 062706. 10.1103/PhysRevE.90.062706 25615128

[B23] Eckberg D. L. (1983). Human sinus arrhythmia as an index of vagal cardiac outflow. J. Appl. Physiol. 54, 961–966. 10.1152/jappl.1983.54.4.961 6853303

[B24] Eckberg D. L. (2003). The human respiratory gate. J. Physiol. 548, 339–352. 10.1113/jphysiol.2002.037192 12626671 PMC2342859

[B25] Elstad M. O’Callaghan E. L. Smith A. J. Ben-Tal A. Ramchandra R. (2018). Cardiorespiratory interactions in humans and animals: Rhythms for life. Am. J. Physiol. 315, H6–H17. 10.1152/ajpheart.00701.2017 29522373

[B26] Friedman L. Dick T. E. Jacono F. J. Loparo K. A. Yeganeh A. Fishman M. (2012). Cardio-ventilatory coupling in young healthy resting subjects. J. Appl. Physiol. 112, 1248–1257. 10.1152/japplphysiol.01424.2010 22267392 PMC3331590

[B27] Galletly D. C. Larsen P. D. (1997). Cardioventilatory coupling during anaesthesia. Br. J. Anaesth. 79, 35–40. 10.1093/bja/79.1.35 9301386

[B28] Galletly D. Larsen P. (1999). Ventilatory frequency variability in spontaneously breathing anaesthetized subjects. Br. J. Anaesth. 83, 552–563. 10.1093/bja/83.4.552 10673869

[B29] Gilbey M. P. Jordan D. Richter D. W. Spyer K. M. (1984). Synaptic mechanisms involved in the inspiratory modulation of vagal cardio-inhibitory neurones in the cat. J. Physiol. 356, 65–78. 10.1113/jphysiol.1984.sp015453 6520798 PMC1193152

[B30] Guzzetti S. Borroni E. Garbelli P. E. Ceriani E. Della Bella P. Montano N. (2005). Symbolic dynamics of heart rate variability: A probe to investigate cardiac autonomic modulation. Circulation 112, 465–470. 10.1161/CIRCULATIONAHA.104.518449 16027252

[B31] Hayano J. Yasuma F. Okada A. Mukai S. Fujinami T. (1996). Respiratory sinus arrhythmia. A phenomenon improving pulmonary gas exchange and circulatory efficiency. Circulation 94, 842–847. 10.1161/01.cir.94.4.842 8772709

[B32] Hirsch J. A. Bishop B. (1981). Respiratory sinus arrhythmia in humans: How breathing pattern modulates heart rate. Am. J. Physiol. 241, H620–H629. 10.1152/ajpheart.1981.241.4.H620 7315987

[B33] Kabir M. M. Saint D. A. Nalivaiko E. Abbott D. Voss A. Baumert M. (2011). Quantification of cardiorespiratory interactions based on joint symbolic dynamics. Ann. Biomed. Eng. 39, 2604–2614. 10.1007/s10439-011-0332-3 21618043

[B34] Kay S. M. Marple S. L. (1981). Spectrum analysis: A modern perspective. Proc. IEEE 69, 1380–1419. 10.1109/proc.1981.12184

[B35] Kuhnhold A. Schumann A. Y. Bartsch R. P. Ubrich R. Barthel P. Schmidt G. (2017). Quantifying cardio-respiratory phase synchronization - a comparison of five methods using ECGs of post-infarction patients. Physiol. Meas. 38, 925–939. 10.1088/1361-6579/aa5dd3 28151433

[B36] Larsen P. D. Tzeng Y. C. Sin P. Y. W. Galletly D. C. (2010). Respiratory sinus arrhythmia in conscious humans during spontaneous respiration. Respir. Physiol. Neurobiol. 174, 111–118. 10.1016/j.resp.2010.04.021 20420940

[B37] Li Z. Ouyang G. Li D. Li X. (2011). Characterization of the causality between spike trains with permutation conditional mutual information. Phys. Rev. E 84, 021929. 10.1103/PhysRevE.84.021929 21929040

[B38] Martini M. Kranz T. A. Wagner T. Lehnertz K. (2011). Inferring directional interactions from transient signals with symbolic transfer entropy. Phys. Rev. E 83, 011919. 10.1103/PhysRevE.83.011919 21405725

[B39] Mazzucco C. E. Marchi A. Bari V. De Maria B. Guzzetti S. Raimondi F. (2017). Mechanical ventilatory modes and cardioventilatory phase synchronization in acute respiratory failure patients. Physiol. Meas. 38, 895–911. 10.1088/1361-6579/aa56ae 28052047

[B40] Moser M. Voica M. Kenner T. Lehofer M. Egner S. Hildebrandt G. (1995). Phase- and frequency coordination of cardiac and respiratory function. Biol. Rhythm Res. 26, 100–111. 10.1080/09291019509360328

[B41] Ottolina D. Cairo B. Fossali T. Mazzucco C. Castelli A. Rech R. (2023). Cardiorespiratory coupling in mechanically ventilated patients studied via synchrogram analysis. Med. Biol. Eng. Comput. 61, 1329–1341. 10.1007/s11517-023-02784-4 36698031

[B42] Palus M. (1997). Detecting phase synchronization in noisy systems. Phys. Lett. A 235, 341–351. 10.1016/s0375-9601(97)00635-x

[B43] Patwardhan A. R. Evans J. M. Bruce E. N. Eckberg D. L. Knapp C. F. (1995). Voluntary control of breathing does not alter vagal modulation of heart rate. J. Appl. Physiol. 78, 2087–2094. 10.1152/jappl.1995.78.6.2087 7665403

[B44] Penzel T. Kantelhardt J. W. Bartsch R. P. Riedl M. Kraemer J. F. Wessel N. (2016). Modulations of heart rate, ECG, and cardio-respiratory coupling observed in polysomnography. Front. Physiol. 7, 460. 10.3389/fphys.2016.00460 27826247 PMC5078504

[B45] Perrott M. H. Cohen R. J. (1996). An efficient approach to ARMA modeling of biological systems with multiple inputs and delays. IEEE Trans. Biomed. Eng. 43, 1–14. 10.1109/10.477696 8567000

[B46] Pinna G. D. Maestri R. La Rovere M. T. Gobbi E. Fanfulla F. (2006). Effect of paced breathing on ventilatory and cardiovascular variability parameters during short-term investigations of autonomic function. Am. J. Physiol. 290, H424–H433. 10.1152/ajpheart.00438.2005 16155106

[B47] Porta A. Bari V. Bassani T. Marchi A. Pistuddi V. Ranucci M. (2013a). Model-based causal closed loop approach to the estimate of baroreflex sensitivity during propofol anesthesia in patients undergoing coronary artery bypass graft. J. Appl. Physiol. 115, 1032–1042. 10.1152/japplphysiol.00537.2013 23869064

[B48] Porta A. Bari V. Gelpi F. Cairo B. De Maria B. Tonon D. (2023). On the different abilities of cross-sample entropy and K-Nearest-Neighbor cross-unpredictability in assessing dynamic cardiorespiratory and cerebrovascular interactions. Entropy 25, 599. 10.3390/e25040599 37190390 PMC10137562

[B49] Porta A. Baselli G. Rimoldi O. Malliani A. Pagani M. (2000a). Assessing baroreflex gain from spontaneous variability in conscious dogs: Role of causality and respiration. Am. J. Physiol. 279, H2558–H2567. 10.1152/ajpheart.2000.279.5.H2558 11045994

[B50] Porta A. Bassani T. Bari V. Pinna G. D. Maestri R. Guzzetti S. (2012a). Accounting for respiration is necessary to reliably infer Granger causality from cardiovascular variability series. IEEE Trans. Biomed. Eng. 59, 832–841. 10.1109/TBME.2011.2180379 22194232

[B51] Porta A. Bassani T. Bari V. Tobaldini E. Takahashi A. C. M. Catai A. M. (2012b). Model-based assessment of baroreflex and cardiopulmonary couplings during graded head-up tilt. Comput. Biol. Med. 42, 298–305. 10.1016/j.compbiomed.2011.04.019 21621756

[B52] Porta A. Baumert M. Cysarz D. Wessel N. (2015a). Enhancing dynamical signatures of complex systems through symbolic computation. Phil. Trans. R. Soc. A 373, 20140099. 10.1098/rsta.2014.0099 25548265 PMC4281870

[B53] Porta A. Castiglioni P. Di Rienzo M. Bassani T. Bari V. Faes L. (2013b). Cardiovascular control and time domain granger causality: Insights from selective autonomic blockade. Phil. Trans. R. Soc. A 371, 20120161. 10.1098/rsta.2012.0161 23858489

[B54] Porta A. Guzzetti S. Montano N. Furlan R. Pagani M. Malliani A. (2001). Entropy, entropy rate and pattern classification as tools to typify complexity in short heart period variability series. IEEE Trans. Biomed. Eng. 48, 1282–1291. 10.1109/10.959324 11686627

[B55] Porta A. Guzzetti S. Montano N. Pagani M. Somers V. Malliani A. (2000b). Information domain analysis of cardiovascular variability signals: Evaluation of regularity, synchronisation and co-ordination. Med. Biol. Eng. Comput. 38, 180–188. 10.1007/BF02344774 10829411

[B56] Porta A. Marchi A. Bari V. Heusser K. Tank J. Jordan J. (2015b). Conditional symbolic analysis detects nonlinear influences of respiration on cardiovascular control in humans. *Phil. Trans. R. Soc.* A 373, 20140096. 10.1098/rsta.2014.0096 25548269 PMC4281867

[B57] Porta A. Takahashi A. C. M. Catai A. M. (2016). Cardiovascular coupling during graded postural challenge: Comparison between linear tools and joint symbolic analysis. Braz. J. Phys. Ther. 20, 461–470. 10.1590/bjpt-rbf.2014.0179 27878227 PMC5123266

[B58] Reulecke S. Charleston-Villalobos S. González-Hermosillo J. González-Camarena R. Voss A. Gaitán-González M. (2018). Study of impaired cardiovascular and respiratory coupling during orthostatic stress based on joint symbolic dynamics. Med. Eng. Phys. 61, 51–60. 10.1016/j.medengphy.2018.08.006 30270005

[B59] Reulecke S. Schulz S. Voss A. (2012). Autonomic regulation during quiet and active sleep states in very preterm neonates. Front. Physiol. 3, 61. 10.3389/fphys.2012.00061 22514535 PMC3322524

[B60] Saul P. J. Berger R. D. Hui Chen M. Cohen R. J. (1989). Transfer function analysis of autonomic regulation II. Respiratory sinus arrhythmia. Am. J. Physiol. 256, H153–H161. 10.1152/ajpheart.1989.256.1.H153 2912177

[B61] Schäfer C. Rosenblum M. G. Abel H. H. Kurths J. (1999). Synchronization in the human cardiorespiratory system. Phys. Rev. E 60, 857–870. 10.1103/physreve.60.857 11969830

[B62] Schäfer G. Rosenblum M. G. Kurths J. Abel H. H. (1998). Heartbeat synchronized with ventilation. Nature 392, 239–240. 10.1038/32567 9521318

[B63] Schreiber T. Schmitz A. (1996). Improved surrogate data for nonlinearity tests. Phys. Rev. Lett. 77, 635–638. 10.1103/PhysRevLett.77.635 10062864

[B64] Schulz S. Adochiei F. C. Edu I. R. Rico S. Hariton C. Bär K. J. (2013a). Cardiovascular and cardiorespiratory coupling analyses: A review. Phil. Trans. R. Soc. A 371, 20120191. 10.1098/rsta.2012.0191 23858490

[B65] Schulz S. Bär K. J. Voss A. (2015). Analyses of heart rate, respiration and cardiorespiratory coupling in patients with schizophrenia. Entropy 17, 483–501. 10.3390/e17020483

[B66] Schulz S. Haueisen J. Bär K. J. Voss A. (2018). Multivariate assessment of the central-cardiorespiratory network structure in neuropathological disease. Physiol. Meas. 39, 074004. 10.1088/1361-6579/aace9b 29933248

[B67] Schulz S. Tupaika N. Berger S. Haueisen J. Bär K. J. Voss A. (2013b). Cardiovascular coupling analysis with high-resolution joint symbolic dynamics in patients suffering from acute schizophrenia. Physiol. Meas. 34, 883–901. 10.1088/0967-3334/34/8/883 23859938

[B68] Skytioti M. Elstad M. (2022). Respiratory sinus arrhythmia is mainly driven by central feedforward mechanisms in healthy humans. Front. Physiol. 13, 768465. 10.3389/fphys.2022.768465 35874518 PMC9301041

[B69] Staniek M. Lehnertz K. (2008). Symbolic transfer entropy. Phys. Rev. Lett. 100, 158101. 10.1103/PhysRevLett.100.158101 18518155

[B70] Suhrbier A. Riedl M. Malberg H. Penzel T. Bretthauer G. Kurths J. (2010). Cardiovascular regulation during sleep quantified by symbolic coupling traces. Chaos 20, 045124. 10.1063/1.3518688 21198136

[B71] Task Force of the European Society of Cardiology and the North American Society of Pacing and Electrophysiology (1996). Heart rate variability: Standards of measurement, physiological interpretation and clinical use. Circulation 93, 1043–1065. 10.1161/01.cir.93.5.1043 8598068

[B72] Triedman J. K. Perrott M. H. Cohen R. J. Saul J. P. (1995). Respiratory sinus arrhythmia: Time domain characterization using autoregressive moving average analysis. Am. J. Physiol. 268, H2232–H2238. 10.1152/ajpheart.1995.268.6.H2232 7611472

[B73] Tzeng Y. C. Larsen P. D. Galletly D. C. (2003). Cardioventilatory coupling in resting human subjects. Exp. Physiol. 88, 775–782. 10.1113/eph8802606 14603377

[B74] Wessel N. Suhrbier A. Riedl M. Marwan N. Malberg H. Bretthauer G. (2009). Detection of time-delayed interactions in biosignals using symbolic coupling traces. EPL 87, 10004. 10.1209/0295-5075/87/10004

[B75] Yana K. Saul J. P. Berger R. D. Perrott M. H. Cohen R. J. (1993). A time domain approach for the fluctuation analysis of heart rate related to instantaneous lung volume. IEEE Trans. Biomed. Eng. 40, 74–81. 10.1109/10.204773 8468078

